# Usefulness and Prospects of Sentinel Lymph Node Biopsy for Patients With Breast Cancer Using the Medical Imaging Projection System

**DOI:** 10.3389/fonc.2021.674419

**Published:** 2021-05-26

**Authors:** Yuki Nakamura, Masahiro Takada, Michiko Imamura, Akane Higami, He Jiaxi, Makoto Fujino, Rie Nakagawa, Yukiko Inagaki, Yoshiaki Matsumoto, Kosuke Kawaguchi, Masahiro Kawashima, Eiji Suzuki, Masakazu Toi

**Affiliations:** ^1^ Department of Surgery (Breast Surgery), Kyoto University Hospital, Kyoto, Japan; ^2^ Department of Breast and Endocrine Surgery, Hyogo College of Medicine, Hyogo, Japan

**Keywords:** breast cancer, sentinel lymph node biopsy, indocyanine green fluorescence method, projection mapping, identification rate

## Abstract

**Background:**

The Medical Imaging Projection System (MIPS) projects indocyanine green (ICG) fluorescence images directly on the surgical field using a projection mapping technique. We conducted an observational study of sentinel lymph node (SLN) biopsy using the prototype MIPS; we found a high identification rate. However, the number of SLN-positive cases was small, and the sensitivity could not be evaluated. The aim of this study was to investigate the clinical usefulness of the MIPS assisted ICG fluorescence method using commercially available equipment.

**Methods:**

This was a retrospective observational study. Patients with primary breast cancer who underwent SLN biopsy using the MIPS at Kyoto University Hospital from April to December 2020 were included in the study. The primary endpoints were the identification rate of SLNs and detection of positive SLNs by the MIPS. The secondary endpoint was the number of SLNs excised using the MIPS per patient. We also conducted a questionnaire survey focused on the utility of the MIPS; it involved doctors with an experience in using the MIPS.

**Results:**

Seventy-nine patients (84 procedures) were included in the study. In 60 (71%) procedures, both the radioisotope (RI) method and MIPS were used. At least one SLN could be detected by the MIPS in all the procedures, with an identification rate of 100% (95% confidence interval 95.6–100%). A total of 19 (7%) positive SLNs were removed, which were identifiable by the MIPS. Among 57 patients in whom the MIPS and RI methods were used, there was no positive SLN only identified by the RI method. The results of the questionnaire survey showed that the MIPS enabled the operator and assistant to share the ICG fluorescence image in the surgical field and to communicate with each other easily.

**Conclusion:**

The current study demonstrated that the identification rate of SLNs using the MIPS was high, and the MIPS can be used for detecting positive SLNs. It was suggested that the MIPS will be useful in learning SLN biopsy procedures.

## Introduction

Sentinel lymph node (SLN) biopsy is the standard of care for patients with clinically node-negative breast cancer. In addition to the radioisotope (RI) and blue dye methods, SLN identification with indocyanine green (ICG) fluorescence was first reported in 2005 (1). From a prior meta-analysis, the SLN identification rates using the ICG fluorescence method and RI method were similar (2).

The Medical Imaging Projection System (MIPS) is a novel near infrared (NIR) fluorescence imaging system that projects ICG fluorescence images directly on the surgical field using a projection mapping technique. The MIPS assisted ICG fluorescence method involves an accurate and continuous projection of ICG fluorescence signals, which enables a real-time navigation surgery for SLN biopsy without shifting the visual focus from the surgical field. It does not require operating lights. In 2018, we conducted an observational study of SLN biopsy using the prototype MIPS in 56 patients (59 procedures) at our institution; the identification rate was comparable to that obtained with the ICG fluorescence method using a conventional NIR system. However, the low percentage of patients with SLN metastasis led to insufficient evaluation of detection of positive SLNs by the MIPS (3).

The aim of this study was to assess SLN identification and detection of positive SLNs using the commercial-type MIPS. We also conducted a questionnaire survey on the utility of the MIPS.

## Patients and Methods

### Patients

The eligibility criteria included: histologically confirmed breast cancer, clinically node-negative tumor, and patients who underwent SLN biopsy using the MIPS. Patients with a previous history of axillary surgery were excluded. Patients who received preoperative systemic therapy (PST) were included.

The study protocol was approved by the institutional review board of Kyoto University Hospital.

### Methods

This was a retrospective observational study. Data on age, body mass index (BMI), tumor size, tumor histology, subtype, with or without PST, number of SLNs, and combined use of the RI method were extracted from the medical records. We also conducted a questionnaire survey involving doctors at Kyoto University Hospital and the Hospital of Hyogo College of Medicine with experience in using the MIPS.

**Figure 1:**
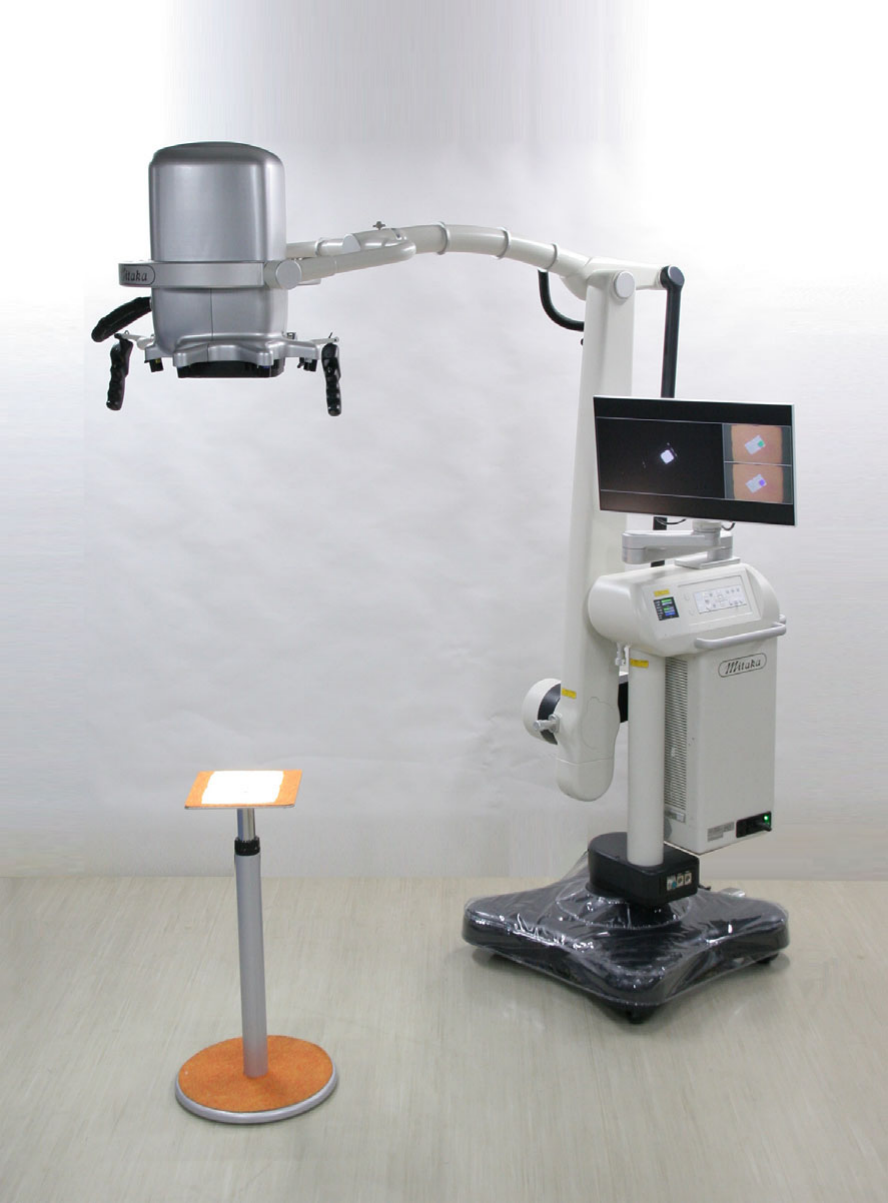
The Medical Imaging Projection System (MIPS). Photograph of the commercial-type MIPS used in this study.

**Figure 2:**
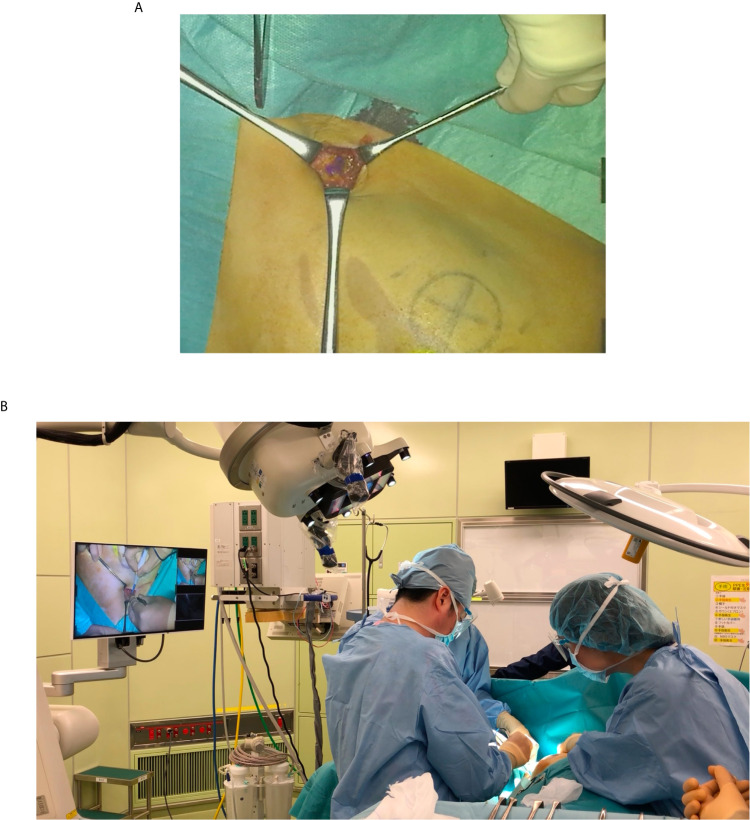
Intraoperative photograph of using the MIPS. **(A)** Operator can confirm the ICG fluorescence image directly in the surgical field. **(B)** Both the operator and assistant can share the ICG fluorescence image in the surgical field.

Detailed features of MIPS and its surgical procedures have been reported previously (4). The commercial-type MIPS was used in this study, in which flexibility of the projection head was improved by introducing the same arm control technology as the surgical microscope; surgeons were able to adjust the projection angle freely during the procedures (**Figures 1** and **2**).

### Endpoints

The primary endpoints included the identification rate of SLNs and detection of positive SLNs by the MIPS. The identification rate was calculated as the percentage of all patients in whom at least one SLN was detected using the MIPS. The detection of positive SLNs was evaluated using the percentage of positive SLNs among all the excised SLNs.

The secondary endpoints included: the number of SLNs excised by the MIPS per patient, relationship between clinical factors and number of detected SLNs, and the detection of SLNs by the MIPS and the RI method among patients after PST. All statistical analyses were performed using JMP^®^ Pro (ver. 15.2.0; SAS Institute, Inc. Cary, NC, USA).

## Results

A total of 79 patients (74 and 5 with unilateral and bilateral tumors, respectively) underwent SLN biopsy using the MIPS from April to December 2020. The analysis was based on 84 procedures. The median age was 59 (range 35–84) years, and the median BMI was 21.1 (range 16.4–32.6) kg/m^2^. More than half of the procedures were performed for T1 tumors (55%), and 15 (18%) procedures were performed for ductal carcinomas *in situ*. Of the 15 patients with DCIS, 13 patients underwent mastectomy and 2 patients were suspected of having invasive carcinoma by imaging findings. Of the 84 procedures, 11 (13%) were performed after PST and all of these patients were clinical node negative. The RI and MIPS methods were both used in 60 (71%) procedures of 57 patients (**Table 1**).

**Table 1:** Patient and tumor characteristics.

Factors	N	%
All procedures	84	
Age, median (range)	59 (35–84)	
Body Mass Index, median (range)	21.1 (16.4-32.6)	
Tumor stage		
Tis	15	18
T1a	6	7
T1b	10	12
T1c	30	36
T2	21	25
T3	1	1
Histology		
Ductal Carcinoma in situ	15	18
Invasive carcinoma (no special type)	60	71
Invasive lobular carcinoma	5	6
Others	4	5
Subtype		
Hormone-receptor-positive and HER2-negative	53	63
Hormone-receptor-positive and HER2-positive	6	7
Hormone-receptor-negative and HER2-positive	2	2
Triple negative	8	10
Unknown	15	18
Histological grade		
1	16	19
2	30	36
3	23	27
Unknown	15	18
Preoperative systemic therapy		
Yes	11	13
No	73	87
Combined use of radioisotope method		
Yes	60	71
No	24	29

HER2, human epidermal growth factor receptor 2.

The identification rate of the MIPS was 100% (84/84: 95% confidence interval [CI] 95.6–100%). Macrometastases were found in 19 (7%) of the 256 SLNs, detected by the MIPS.

Of 185 SLNs excised among the patients who underwent SLN biopsy using both the MIPS and RI methods, 90 (49%) SLNs were identified by both the MIPS and RI methods, 91 (49%) only by the MIPS, 2 (1%) only by the RI method, and 2 (1%) by neither the MIPS nor RI method. The number of positive SLNs detected by both the MIPS and RI methods was 8, that only by the MIPS was 1, that only by the RI method and by neither the MIPS nor RI method were 0 (**Table 2**).

**Table 2:** The number of SLNs among cases using MIPS and RI.

SLN biopsy procedures	Identified SLNs (total N=185)	Positive SLNs(total N=9)
MIPS and RI	90	8
MIPS only	91	1
RI only	2	0
Neither MIPS nor RI (Palpation)	2	0

SLN, sentinel lymph node; MIPS, medical imaging projection system; RI, radioisotope.

The median number of SLNs detected by the MIPS per patient was 3 (range, 1–6). The median number of SLNs identified by the MIPS among patients with a high BMI (≥ 22 kg/m^2^) was almost equal to that of patients with a low BMI (<22 kg/m^2^) (*P* = 0.09). Of the procedures performed after PST, the median number of SLNs identified by the MIPS was 3 (range, 2–5) (**Table 3**).

**Table 3:** Number of SLNs identified by the MIPS according to patient characteristics.

Characteristics	N	Median	Range	P value
All procedures	84	3	1-6	
BMI				
<22 kg/m^2^	46	3	1-6	0.09
≧22 kg/m^2^	38	2.5	1-5	
PST				
Yes	11	3	2-5	0.75
No	73	3	1-6	

SLN, sentinel lymph node; BMI, body mass index; PST, preoperative systemic therapy.

Among patients who underwent SLN biopsy after PST, the identification rate of the MIPS was 100% (95% CI 74.1-100%) and that of the RI method was 100% (95% CI 70.1-100%). The median number of SLNs identified by the RI method was 1 (range, 1-3). The number of positive SLNs was one, which was detected by both the MIPS and RI methods.

The results of the questionnaire survey are shown in **Table 4**. Regarding the clinical advantage of the MIPS, 69% of the doctors answered that operators could perform the procedures easily by confirming the ICG fluorescence image directly in the surgical field; 54% answered that operators could perform the procedures without disrupting the surgical workflow since the MIPS did not require operating lights during the procedures; 62% answered that operators could operate smoothly without holding the NIR camera, and 62% answered that both the operator and assistant could communicate with each other easily by sharing the ICG fluorescence image in the surgical field during the surgery. Regarding the relationship between the MIPS and procedure difficulty including duration of surgery, 38% of the doctors felt that the MIPS led to a shorter duration of surgery, 31% did not feel so, and 31% were indifferent.

**Table 4:** Results of the questionnaire survey for doctors with an experience in using MIPS.

Questionnaire survey	Number of ‘yes’	% (total 13)
Which do you consider to be a clinical advantage of MIPS?		
Operators can perform the procedures easily by confirming the ICG fluorescence image directly in the surgical field.	9	69
Operators can perform the surgery without disruption of the surgical workflow since MIPS does not require operating lights.	7	54
Operators can operate smoothly without holding the NIR camera.	8	62
The operator and assistant can communicate with each other easily due to the possibility of sharing the ICG fluorescence image.	8	62
Do you feel the MIPS leads to shorter surgery duration?		
Yes	5	38
No	4	31
Neither	4	31

MIPS, medical imaging system projection system; ICG, indocyanine green.

## Discussion

The current study showed the reproducibility of the MIPS for SLN identification and the feasibility of detection of positive SLNs by the MIPS.

It has been reported that the SLN identification rate ranged from 89–100%, and the mean number of removed SLNs was 1.5–3.4, using the ICG fluorescence method (2). In the current study, the SLN identification rate was 100% (95% CI: 95.6–100%), and the median number of SLNs detected by MIPS was 3 (range 1–6), and our results were comparable to those of the ICG fluorescence method using a conventional NIR system.

In this study, 17% of the patients had positive SLNs, which was higher than that in our previous study; however, it was lower than those in previous reports (5). Preoperative examination of axillary lymph nodes in our institution includes palpation, ultrasonography, and contrast-enhanced magnetic resonance imaging (CE-MRI). If nodal involvement is suspected, ultrasound-guided fine-needle aspiration biopsy (US-FNA) is also performed. CE-MRI and US-FNA increase the accuracy of preoperative axillary staging (4, 6); the involvement of these examinations may have contributed to the low proportion of positive SLNs in our study. In this study, there was no positive SLN that was identified only by the RI method, and the MIPS identified all the positive SLNs in the patients in whom both the RI method and the MIPS were used. It is suggested that the detection of positive SLNs by the MIPS is comparable to detection by the RI method.

It has been reported that the number of detected SLNs decreases as the BMI increases (7). However, in our study, the number of SLNs excised from patients with a high BMI was almost equal to that from patients with a low BMI. The MIPS may be useful in identifying SLNs among patients with a high BMI. We used BMI 22 as cutoff value, following the domestic standard value in Japan. There was no statistically significant difference in the number of sentinel lymph node identified, when we used the cutoff value of 25, 28 and 30 (p value 0.33, 0.75 and 0.62, respectively).

SLN biopsy after PST is feasible for patients with clinically node-negative cancer at baseline (8, 9). However, SLN biopsy after PST for patients with clinically node-positive cancer has a low identification rate and a high false-negative rate (10–12). Removal of three or more SLNs has been reported to reduce the false-negative rate to below 10% in patients with clinically node-positive cancer (13). In the present study, SLN biopsies after PST were performed in 11 (13%) procedures. Although this is a small number of cases, the identification rate by the MIPS among them was 100% (95% CI 74.1–100%), and the median number of excised SLNs was 3 (range 2–5).

The main clinical advantage of the MIPS is the ease of communication between the operator and assistant due to the possibility of sharing the ICG fluorescence image in the surgical field. From the results of the questionnaire survey, more than half of the doctors confirmed this advantage. The percentage of doctors who considered the communication between operator and assistant as a clinical advantage of the MIPS was 100%, 25%, and 67% among doctors who graduated ≤5 years, 6–10 years, and ≥11 years prior to the procedures. This tendency indicated that the communication between the operator and assistant would be useful in teaching the procedures to doctors willing to learn. We also expected a shorter duration of surgery with the MIPS, but the percentage of doctors who felt that using the MIPS shortened the duration of surgery was only 38%.

There are several limitations to the present study. First, our sample size was relatively small; however, it did not hinder the clinical utility of the MIPS, as shown by the lower limit of the 95% CIs, which was above 90%. Second, the retrospective nature of the study; however, our results were comparable to those of our previous prospective observational study. Third, the small number of SLN biopsies after PST; we need to investigate the utility of the MIPS in more cases after PST. Forth, the evaluation of the MIPS in learning procedures was not objective; we need to evaluate the learning curve of SLN biopsy procedures using the MIPS.

In conclusion, this study demonstrated that the identification rate of SLNs using the MIPS was high and the MIPS can be used for the detection of positive SLNs. The MIPS enabled an ease of communication between the operator and assistant. It was suggested that this advantage of the MIPS will be useful in learning SLN biopsy procedures.

## Data Availability Statement

The original contributions presented in the study are included in the article/supplementary material. Further inquiries can be directed to the corresponding author.

## Ethics Statement

The studies involving human participants were reviewed and approved by Kyoto University Graduate School and Faculty of Medicine, Ethics Committee. Written informed consent for participation was not required for this study in accordance with the national legislation and the institutional requirements.

## Author Contributions

YN contributed to data collection and analysis and wrote this manuscript. MTa made important corrections to the manuscript. MI, AH, HJ, MF, RN, YI, YM, KK, MK, ES, and MTo answered the questionnaire survey and checked the final version of the manuscript. All authors contributed to the article and approved the submitted version.

## Conflict of Interest

The authors declare that the research was conducted in the absence of any commercial or financial relationships that could be construed as a potential conflict of interest.
